# The Treatment of Leprosy

**Published:** 1901-05-11

**Authors:** 


### THE TREATMENT OF LEPROSY.

Not many cases of leprosy recover, and when this fortunate result does happen it is generally difficult to be sure how much of the credit is due to hygiene and how much to medicine. Dr. George Thin,[1] however, records two cases in which he thinks he may fairly attribute the cure to the action of drugs. The first was a boy aged 11 years who was suffering from fully-developed and severe nerve leprosy which had been going on for about seven years. The legs and thighs were discoloured and completely anæsthetic over a large extent of surface. The fingers of both hands were "clawed" and some mutilation had taken place. There was anæsthesia about the arms and wrists, and also on the face, which was partially discoloured. The eyes were watery and bleared, and the eyebrows were defective. The boy had the physique of a lad of seven or eight instead of eleven, the limbs being small and shrunken and his appearance that of an atrophied and wasted creature. The main line of treatment adopted was the use of chaulmoogra oil internally and externally. After three months of treatment his general condition was much improved. He took the oil for between two and three years, during which time he greatly improved. In the end he recovered, and when seen thirteen years later he was quite well, as he had been for a number of years. Dr. Thin attributes the recovery to the use of the chaulmoogra oil, because before he began to take it he had been steadily deteriorating, and although hygienic measures may have contributed to the result, it must be borne in mind that he had always been well cared for and that his family (English people of the professional class) were in comfortable circumstances. The second case was a youth in whom nerve leprosy was detected at a very early stage, the lesion consisting of a round, slightly discoloured patch on the face which had existed for six months. The appearance was characteristic of nerve leprosy in the early stage. On carefully examining the rest of the body a similar patch, scarcely larger than a threepenny piece, was found over the spine between the shoulders. Both of the patches were anæsthetic. The treatment prescribed in this case was 5 per cent. pyrogallic acid ointment, to be rubbed into both places twice daily, a drachm dose of gurgun oil to be taken twice a day, and two minims of Fowler's solution twice daily. The ointment was afterwards increased in strength to 7½ per cent. The small patch between the shoulders entirely disappeared in less than a month, and perfect sensation was restored. About a year later several small, pale, anæsthetic spots, not larger than a split pea, appeared on one of the thighs, and two brown patches on the calf, but they all cleared up under the pyrogallic ointment. The ointment and the gurgun oil were continued for more than two years, but the arsenic could only be taken intermittently. In the end recovery was complete, and since then the patient has led an active, useful life, enjoying excellent health. Dr. Thin is convinced that in this case the pyrogallic acid ointment eradicated the small patches of anæsthetic leprosy, and he specially calls attention to this treatment, for which we have to thank Dr. Unna.

[1] Brit. Med. Journ., May 4.

